# High-Temperature Dielectric Properties of Aluminum Nitride Ceramic for Wireless Passive Sensing Applications

**DOI:** 10.3390/s150922660

**Published:** 2015-09-08

**Authors:** Jun Liu, Yukun Yuan, Zhong Ren, Qiulin Tan, Jijun Xiong

**Affiliations:** 1 Science and Technology on Electronic Test & Measurement Laboratory, North University of China, Taiyuan 030051, China; E-Mails: Liuj@nuc.edu.cn (J.L.); yuanyukun_ever@163.com (Y.Y.); rz381567720@163.com (Z.R.); 2 Key Laboratory of Instrumentation Science & Dynamic Measurement, Ministry of Education, North University of China, Taiyuan 030051, China

**Keywords:** wireless passive, aluminum nitride ceramic, LC resonator, dielectric properties, high temperature, quality factor

## Abstract

The accurate characterization of the temperature-dependent permittivity of aluminum nitride (AlN) ceramic is quite critical to the application of wireless passive sensors for harsh environments. Since the change of the temperature-dependent permittivity will vary the ceramic-based capacitance, which can be converted into the change of the resonant frequency, an LC resonator, based on AlN ceramic, is prepared by the thick film technology. The dielectric properties of AlN ceramic are measured by the wireless coupling method, and discussed within the temperature range of 12 °C (room temperature) to 600 °C. The results show that the extracted relative permittivity of ceramic at room temperature is 2.3% higher than the nominal value of 9, and increases from 9.21 to 10.79, and the quality factor *Q* is decreased from 29.77 at room temperature to 3.61 at 600 °C within the temperature range.

## 1. Introduction

Aluminum nitride (AlN), a new generation of ceramic material, has excellent comprehensive performance for temperature corrosion resistance, stability, high strength, and hardness, which is very promising in the application of high temperature structural materials and electronic industry [1,2]. In addition, the commercial importance of AlN stems from its high thermal conductivity, and relatively low permittivity, which makes it enticing as a substrate material for microelectronic devices, especially suitable for wireless passive sensing applications [3,4,5,6]. The most accurate methods of measuring low loss dielectric materials are resonance methods employing cavity perturbation technique [7,8,9], surface acoustic wave devices [10] or Lamb wave resonators [11], and dielectric resonance technique [12,13,14], mainly based on post resonance, cylinder cavity resonance and waveguide reflection resonance. 

In this paper, we demonstrate a method to measure the dielectric properties of AlN ceramic for wireless passive sensing applications under harsh environments. The LC resonator, integrating a planar spiral inductor and a parallel plate capacitor, is designed and fabricated using the screen-printing and sintering techniques, and the temperature-dependent dielectric properties of AlN ceramic are measured and discussed. The permittivity behavior serves as the temperature sensing mechanism, and the accurate characterization of the permittivity of AlN materials is critical to develop wireless passive sensors for harsh environments, and is of guiding significance to the research of temperature-compensation for pressure sensors under complex environments [15,16].

## 2. Principle of Operation

The change of the temperature-dependent permittivity will vary the ceramic-based capacitance, which can be converted into the change of the resonant frequency. Therefore, we can realize the wireless measurement of ceramic permittivity by the readout of the resonant frequency of a ceramic-based LC resonator at different temperatures. Equivalent circuit model of the resonator is mainly a series resonant circuit, including the series inductance *L_s_*, the series capacitance *C_s_* and the series resistance *R_s_*. The resonant frequency *f*_0_ and quality factor *Q* of the LC circuit are given by [17]:

(1) $$f_{0}=\frac{1}{2\pi \sqrt{L_{s}C_{s}}}$$

(2) $$Q=\frac{2\pi f_{0}}{R_{s}}L_{s}$$

**Figure 1:**
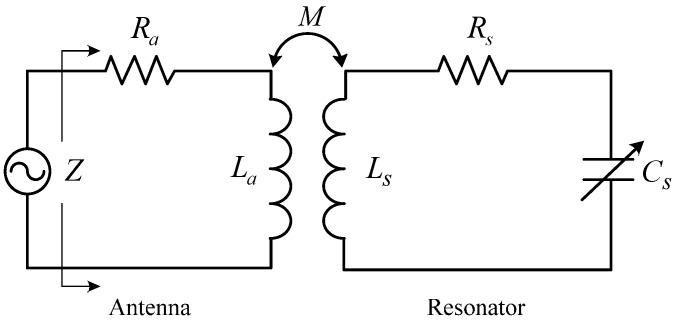
Equivalent circuit.

The resonant frequency of the resonator, varied with external temperatures, can be detected by a read circuit with an antenna in a wireless coupling method, as shown in Figure 1.

Coupling coefficient *k* between the resonator and the antenna is related to mutual inductance *M*, given by:

(3) $$k=\frac{M}{\sqrt{L_{a}L_{s}}}$$

and the total input impedance *Z* looking into the antenna can be concluded by:

(4) $$Z=R_{a}+j2\pi fL_{a}\left[1+\frac{k^{2}{\left(f/f_{0}\right)}^{2}}{1-{\left(f/f_{0}\right)}^{2}+jf/(f_{0}Q)}\right]$$

where *R_a_* is the series resistance of the antenna, *L_a_* and *L_s_* are the series inductance of the antenna and resonator, respectively, and *f* is the sweep-frequency loaded at the terminal of the antenna.

**Figure 2:**
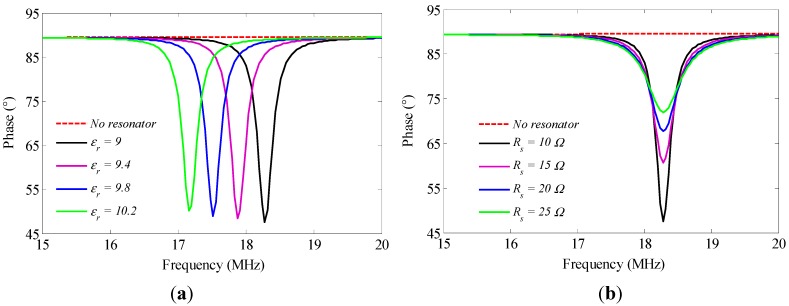
The simulation phase of *Z versus* frequency at different *ε_r_* (**a**) and *R_s_* (**b**).

As we can see from Equation (4), the resonant frequency of the LC resonator can be determined by monitoring the frequency response of the phase of the input impedance *Z*. When the resonator is in the interrogation zone of the antenna, a sharp resonance will appear around the resonant frequency of the resonator, as shown in Figure 2. However, the minimum frequency *f*_min_ is not equal to the resonant frequency of the resonator. The relationship between them can be expressed by the simple equation [18]:

(5) $$f_{\min}=f_{0}\left(1+\frac{k^{2}}{4}+\frac{1}{8Q^{2}}\right)$$

Equation (5) presents a general idea of the relationship between *f*_min_ and the relative permittivity *ε_r_* of aluminum nitride ceramic. The higher *ε_r_* becomes, the lower *f*_min_ gets, and the minimum phase increases slightly. Additionally, an increase of *R_s_* will induce obvious increases of the minimum phase and resonance bandwidth.

## 3. Resonator Design

**Figure 3:**
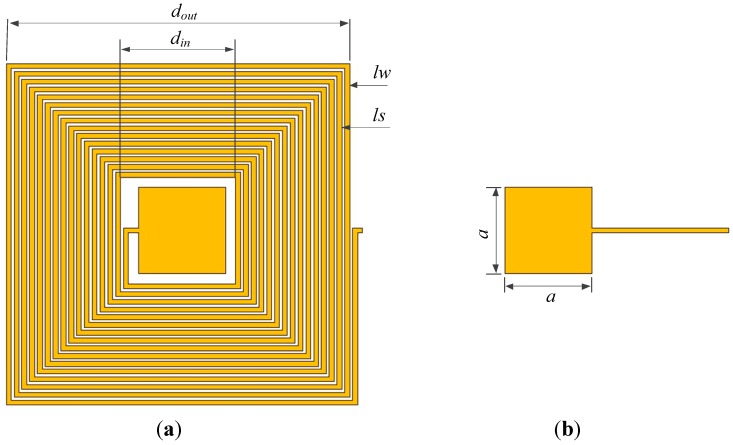
Design parameters of resonator: top electrode and inductance coil (**a**) and bottom electrode (**b**). *d_out_* and *d_in_* are the outer diameter and inner diameter of the inductor, respectively. *lw*, *ls* and *lt* are line width, line spacing and line thickness of the inductor. *a*: length of capacitor plates.

The LC resonator based on AlN ceramic consists of a planar spiral inductor and a parallel electrode plate capacitor, as shown in Figure 3.

### 3.1. Inductance Design

The planar spiral inductor is designed to be square, and the calculation of the inductance is given by [19]:

(6) $$L_{S}=K_{1}\mu_{0}\frac{n^{2}d_{avg}}{1+K_{2}\rho }$$

where *n* means the inductor turns, *ρ* is filling ratio, $\rho =\left(d_{out}-d_{in}\right)/\left(d_{out}+d_{in}\right)$, *d_avg_* corresponds to the average diameter of the inductor, $d_{avg}=\left(d_{in}+d_{out}\right)/2$, the magnetic permeability of free space *μ_0_* is 4π × 10^−7^ H/m, and *K_1_* and *K_2_* are the correlation coefficient, assigned the value of 2.34 and 2.75, respectively.

### 3.2. Capacitance Design

The capacitor plates are also designed to be square, and the calculation of the capacitance is given by [20]:

(7) $$C_{s}(T)=\varepsilon_{r}(T)\frac{\varepsilon_{0}a^{2}}{t_{m}}$$

where *ε_0_* is the permittivity of free space, 8.85 × 10^−12^ F/m, *ε_r_* is the temperature-dependent relative permittivity of alumina ceramic, and *t_m_* is the thickness of the ceramic substrate.

### 3.3. Resistance Estimation

To compute and consider the losses due to proximity, and skin depth related to the distribution of the current density within the conductor, the series resistance of the inductor increases with frequency is modeled through the approximate analytical equation [21]:

(8) $$R_{s}\left(f\right)=R_{dc}\left[1+\frac{1}{10}\left(\frac{f}{f_{crit}}\right)\right]$$

where *R_dc_* denotes the planar spiral inductor’s series resistance at DC and *f_cirt_* is the frequency at which the current crowding begins to become significant. They can be calculated according to:

(9) $$R_{dc}=\rho \frac{ll}{lw\cdot lt}$$

and [18]

(10) $$f_{cirt}=\frac{6.2\cdot \pi \cdot \rho \cdot \left(lw+ls\right)}{\mu_{0}\cdot lt\cdot lw^{2}}$$

here, *ρ* is the sheet resistivity for the planar spiral inductor on aluminum nitride ceramic, estimated to be about 1.27 × 10^−7^ Ω·m, and *ll* is the total length of the inductor trace.

### 3.4. Q and f_0_

It is known that the higher the operating frequency is, the greater the parasitic capacitance and inductance will influence a system. Thus, the design value of the resonance frequency of the resonator should not be designed too high. It can be concluded from Equation (2) that, if the resonator has high inductance, low capacitance, and equivalent series resistance, the resonator will obtain a high Q. Parameters of the resonator are summarized in Table 1. It should be noted that the quality factor Q of the resonator can be further improved by designing a high inductance and a low resistance, and adjusting the shape and geometric dimensions of the inductance coil.

**Table 1:** Design parameters at room temperature (25 °C).

Parameters	Value	Parameters	Value
*n*	15.5	*ε_r_*	9
*d_in_*/mm	11	*C_s_*/pF	12.43
*d_out_*/mm	36	*L_s_*/µH	6.72
*t_m_*/mm	0.508	*L_a_*/µH	~2
*a*/mm	8.9	*R_s_*/Ω	25.9
*lw*/mm	0.5	*R_a_*/Ω	1.5
*ls*/mm	0.3	*f_0_*/MHz	17.4
*lt*/μm	~20	*Q*	28.37

## 4. Fabrication

**Figure 4:**
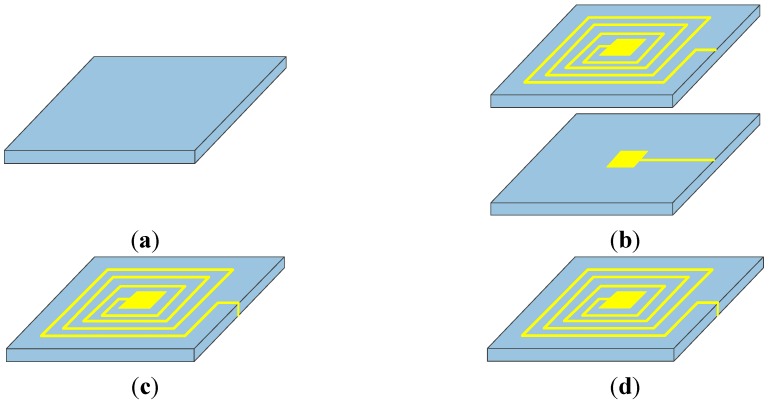
Fabrication process of the resonator. (**a**) Surface pre-process; (**b**) Screen-print circuit: Top electrode and inductance coil and bottom electrode; (**c**) Cure the conductive paste; (**d**) Examine.

AlN ceramic for the experiment is made under atmospheric pressure sintering, and the fabrication process of the LC resonator is illustrated in Figure 4. The substrate is composed of AlN ceramic material (CanaryTec Co., Ltd., Guangzhou, China) and its characteristic parameters are shown in Table 2.

**Table 2:** Characteristic parameters of AlN ceramic.

Parameters	Value
Density (g/cm^3^)	3.335
Flexural Strength (MPa, 25 °C)	330
Thermal conductivity (W·m^−1^·K^−1^, 25 °C)	≥170
CTE (ppm/°C, 20~300 °C)	2.805
Relative permittivity (25 °C, 1 MHz)	9.0
Dielectric loss (25 °C, 1 MHz)	0.0004
Resistivity (MΩ·m, 25 °C)	1.4 × 10^6^

**Figure 5:**
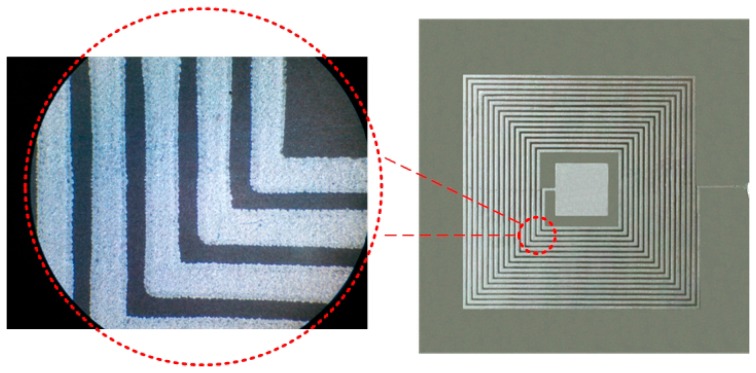
Prototype of the resonator.

The conductive Ag/Pd/Pt paste, ESL 9562-G (ESL Electroscience, UK), is screen-printed on the substrate to form a LC resonant circuit. However, the metallization of AlN ceramic is relatively difficult because the conductive pastes, which have been commercially applied on alumina ceramic, usually cannot be directly used for AlN ceramics. Otherwise, it will weaken the metallic bonding strength, which has been confirmed by Yamaguchi and Kageyama [22]. Therefore, the first step, surface pre-process, is necessary before metallization, as shown in Figure 4 (step 1). Sinters the AlN ceramic in the Nabertherm LHT-02/16 high temperature desktop furnace by heating to 1200 °C and holding for 60 min, which can make the ceramic surface generate alumina in case of a chemical reaction on the surface [23]. At the same time, the chosen conductive paste contains palladium to improve the bonding strength. The next step is to screen-print the conductive paste on the substrate to form the top electrode and inductance coils using screen printing processes, and then the conductive paste on the substrate is dried at 150 °C for 10 min in an infrared oven, as shown in Figure 4 (step 2a). Next, repeat step 2a for the bottom capacitor electrode, as shown in Figure 4 (step 2b). To form a complete closed LC circuit, a scraper is used to spread a line of paste from the external connection point of the inductance coil to the bottom electrode. The third step is to cure the paste in a furnace. The firing ramp rate is 15 °C/min to a peak temperature 850 °C, holding for 15 min, and then removing the metallized aluminum nitride ceramic from the furnace after cooling. Finally, the fabricated resonator needs to be examined for the conduction status of the circuit. A multimeter is used to measure the DC resistance of the circuit in order to determine whether the circuit is open, and a microscope is used to observe an enlarged view of the inductance coil to determine whether it is a short inductor. The prototype of the resonator is presented in Figure 5.

## 5. Experiment and Discussion

**Figure 6:**
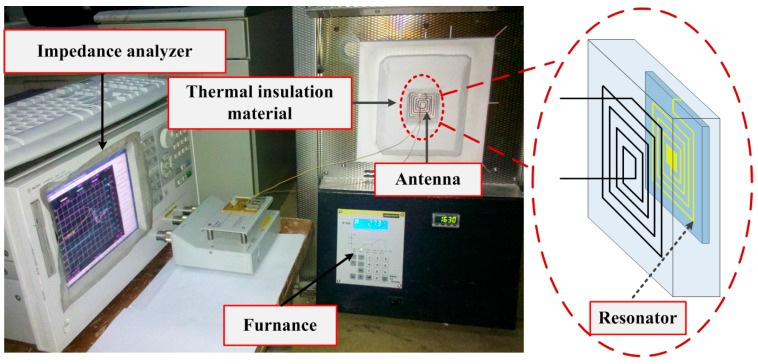
Experiment setup for the LC resonator.

The experimental setup for the LC resonator is shown in Figure 6. The resonator is affixed to the inside of the thermal insulation material, and the antenna is placed in the recess on the outside of the thermal insulation material. The thickness of the insulating layer is 10 mm between the resonator and the antenna, namely to maintain a coupling distance of 10 mm, where the maximum coupling distance is 40 mm. A Nabertherm LHT-02/16 high temperature desktop furnace, used to heat the resonator, ranged from 12 °C (room temperature) to 600 °C, and an Agilent E4991A impedance analyzer is used to analyze the variation of phase of the input impedance according to sweep-frequency signal.

According to Equation (4), curve fitting is used to extract the resonance frequency *f_0_* and quality factor *Q* from the measured data of impedance phase *versus* temperature. The resonance frequency *f_0_* and quality factor *Q* of the resonator are measured to be 17.22 MHz and 29.77, at room temperature, respectively. The former is lower than the design value of 17.4 MHz, which is mainly affected by processing errors and parasitic parameters. The latter is higher than the design value of 28.37, which is likely due to the line thickness of the inductor of more than the design value of 20 µm from the screen-printing process.

**Figure 7:**
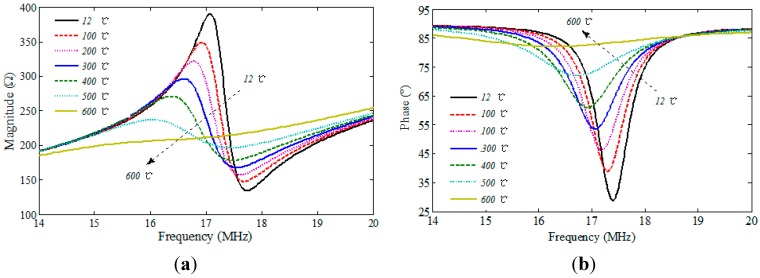
The magnitude (**a**) and phase (**b**) of impedance versus frequency over 12 °C–600 °C temperature range.

The magnitude and phase of the input impedance are changed according to different temperatures, from room temperature to 600 °C, as shown in Figure 7. As can be seen, as the temperature rises, the frequency characteristic curves of the magnitude and the phase are both shifted to the low frequency domain, and the minimum phase and resonance bandwidth increase gradually, which is consistent with the results shown in Figure 2. Thus, it is subject to the combined effect of the permittivity of aluminum nitride ceramic and the series resistance of the LC circuit. Additionally, the coupling strength decreases rapidly, especially with the sharp increase of the resonant bandwidth in the temperature range of 500 °C to 600 °C, and it weakens the coupling strength when the resonator is heated greater than a temperature of 600 °C.

**Figure 8:**
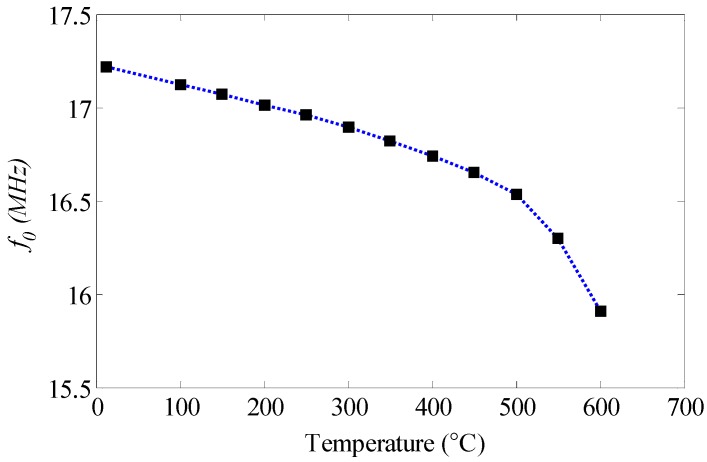
Resonant frequency *versus* Temperature.

The curve of the resonant frequency *versus* temperature is illustrated in Figure 8. The resonant frequency decreases slowly, depending almost linearly on the temperature within the range of room temperature to 500 °C, and the average change rate of the resonance frequency to temperature is about −1.41 kHz/°C. However, the resonant frequency decreases sharply, and the average change rate of the resonance frequency to temperature is up to −6.20 kHz/°C within the temperature range of 500 °C to 600 °C.

**Figure 9:**
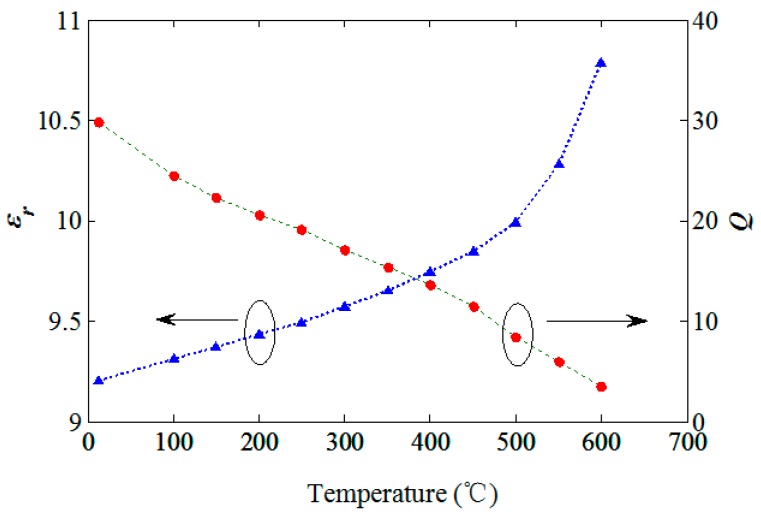
Relative permittivity and Q *versus* temperature.

The inductance at low frequency of planar spiral inductor is mainly dependent on its physical dimensions influenced by the low coefficient of thermal expansion (CTE) of aluminum nitride ceramic, which is used as the substrate of the resonator. It means that inductance is not greatly affected within the temperature range of room temperature to 600 °C. Therefore, the resonant frequency of the resonator decreases due to the increase of capacitance, which is dependent on the increasing permittivity of the ceramic. To illustrate the phenomenon, it obviously indicates a downshift of resonant frequency by the curve of the impedance phase *versus* frequency corresponding to different relative permittivity of ceramic, as shown in Figure 2a. Since the resonant frequency change against temperature is monotonic, the permittivity of aluminum nitride ceramic can be extracted from the measured *f_0_* according to different temperatures. As shown in Figure 9, the extracted relative permittivity of ceramic at room temperature is 2.3% higher than the nominal value of 9, and increases from 9.21 to 10.79 within the temperature range.

The series resonant circuit is mainly made from the conductive paste, using screen-printing technology. When the temperature increases, the resistivity of the paste grows, thereby increasing the equivalent series resistance of the circuit. The bigger *R_s_* is, the smaller the quality factor Q of the resonator is, which matches well with the curve of the impedance phase *versus* frequency corresponding to different series resistance of circuit, as shown in Figure 2b. Figure 9 shows the measured Q of the resonator within the temperature from room temperature to 600 °C. *Q* is decreased from 29.77 at room temperature to 3.61 at 600 °C, which limits the operation range of the resonator fabricated from the aluminum nitride ceramic materials.

Quality factor Q of the resonator is influenced by temperature-dependent losses, mainly including the metal resistivity and dielectric loss, which can be reflected by a resonance bandwidth. To find the key factor influencing Q, the conductivity of another AlN ceramic, without metallization, is measured in contrast with the same size of the alumina ceramic. The results show that the sheet conductivity is about 10 times higher than that of alumina within the temperature range of 500 °C to 600 °C, and the conductivity of AlN ceramic increases rapidly, which is basically consistent with the test results in the article written by Francis [24]. In addition, the alumina ceramic with the same metallization process can still obviously detect the resonance signal when the temperature exceeds 600 °C, though it is very weak for the AlN ceramic. Hence, it can be concluded that the dielectric loss dominates the decreased Q due to increased conductivity. Therefore, Q can be improved at high temperatures if the conductivity of the AlN material has a lower dependence on temperature.

## 6. Conclusions

As the temperature rises, the frequency characteristic curve of the impedance phase is shifted to the low frequency domain, and the minimum phase and resonance bandwidth increase gradually, which is subject to the combined effect of the permittivity of aluminum nitride ceramic and the series resistance of the LC circuit. The average change rate of the resonance frequency to temperature is about −1.41 kHz/°C within the range of room temperature to 500 °C, while it is up to −6.20 kHz/°C within the temperature range of 500 °C to 600 °C. The extracted relative permittivity of ceramic at room temperature is 2.3% higher than the nominal value of 9, and increases from 9.21 to 10.79 within the temperature range, and Q is decreased from 29.77 at room temperature to 3.61 at 600 °C. When the temperature exceeds 600 °C, the resonant bandwidth increased significantly, and the Q value decreases are closely related with the increased conductivity of the AlN ceramic.
